# Determination of lead, cadmium and arsenic in infusion tea cultivated in north of Iran

**DOI:** 10.1186/1735-2746-9-37

**Published:** 2012-12-31

**Authors:** Sakine Shekoohiyan, Mahboobeh Ghoochani, Azita Mohagheghian, Amir Hossein Mahvi, Masoud Yunesian, Shahrokh Nazmara

**Affiliations:** 1 Department of Environmental Research, Center for Social Determinants in Health Promotion, Hormozgan University of Medical Sciences, Bandar-Abbas, Iran; 2 Department of Environmental Health Engineering, School of Public Health, Tehran University of Medical Sciences, Tehran, Iran; 3 National Institute of Health Research, Tehran University of Medical Sciences, Tehran, Iran; 4 Center for Solid Waste Research, Institute for Environmental Research, Tehran University of Medical Sciences, Tehran, Iran

**Keywords:** Heavy metals, Lead, Cadmium, Arsenic, Tea

## Abstract

Tea is one of the most common drinks in all over the world. Rapid urbanization and industrialization in recent decades has increased heavy metals in tea and other foods. In this research, heavy metal contents such as lead (Pb), cadmium (Cd) and arsenic (As) were determined in 105 black tea samples cultivated in Guilan and Mazandaran Provinces in north of Iran and their tea infusions. The amount of heavy metals in black tea infusions were analyzed using Inductively Coupled Plasma Atomic Emission Spectroscopy (ICP - AES).

The mean ± SD level of Pb in 5, 15 and 60 min in infusion tea samples were 0.802 ± 0.633, 0.993 ± 0.667 and 1.367 ± 1.06 mg/kg of tea dry weight, respectively. The mean level of Cd in 5, 15 and 60 min in infusion tea samples were 0.135 ± 0.274, 0.244 ± 0.46 and 0.343 ± 0.473 mg/kg of tea dry weight, respectively. The mean level of As in 5, 15 and 60 min in infusion tea samples were 0.277 ± 0.272, 0.426 ± 0.402 and 0.563 ± 0.454 mg/kg of tea dry weight, respectively. Also, the results showed that the locations and the infusion times influenced upon the amount of these metals (P < 0.05).

## Introduction

Tea is one of the most common drinks in all over the world, which is produced from the leaves of a shrub *Camellia sinensis*[1]. About 98% of people drink tea as the first among all beverages available to use [2]. The 75% of the estimated 2.5 million metric tons of desiccated tea that are produced annually is processed as black tea which is used by many countries [3]. About 18–20 billion cups of tea are consumed daily in the world [4].

Iran is a tea-producing country and relies on substantive imports to supplement its production to satisfy the considerable consumption needs. Iranians hold one of the highest per capita rates in tea consumption in the world, (about1.6 kg per capita consumption in the period of 2005–2007), [5]. Approximately 34 thousand hectares of lands in Guilan and Mazandaran provinces have been cultured for tea, almost half of the dry tea interior production and the remainder comes from imports. Most important source of nutrient uptake by leaf tea is from the medium [4].

Many studies have proved that tea has some benefi-cial effects on human health such as prevention from Parkinson’s disease, cardiovascular disease [6], cancer [7], immune disorders [8] and decrease of blood cholesterol levels [9]. Rapid urbanization and industrialization in recent decades has increased these metals in tea and other foods [10]. Plants receive these trace elements from growth media [11]; other sources include application of pesticides and fertilizers [12]. Heavy metals such as Pb, Cd and As create concerns for tea consumers. In addition, fluoride concentration is usually very high in black tea [13]. Pb is a physiologic and neurological toxin that can affect several organs in the human body. Each country sets tolerable limit for Pb concentration in tea leaves. And 20 mg/kg in Japan, while in China the limit is 2 mg/kg.

Cd and Pb can reduce cognitive development and intellectual performance in children and damage kidneys and the reproductive system [14]. Cd is also identified to be carcinogenic, and has been related to lung and prostate cancer [15]. Arsenic is strongly related with lung and skin cancer in humans, and may cause other interior cancers as well. Skin lesions, peripheral neuropathy, and anemia are characteristics of chronic arsenic intake. Also, As is a major risk factor for black foot disease, which is caused by intake polluted ground water [16]. In a study the concentration of Aluminum was determined in black tea infusion samples [17]. Therefore, determination of these elemental compositions in tea and related products is very essential [18]. The main aim of this study was to investigate the quantity of Pb, As and Cd levels in tea samples that are cultivated in Guilan and Mazandaran provinces in north of Iran.

## Materials and methods

**Figure 1:**
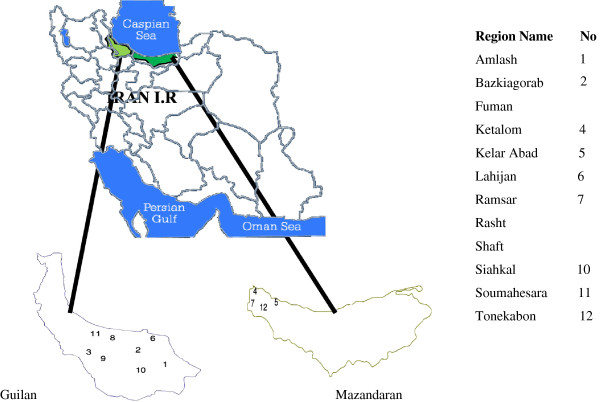
Location of Guilan and Mazandaran provinces and sampling regions.

This study was carried out between September and November 2010 on tea samples that are cultivated in Guilan and Mazandaran Provinces in north of Iran (Figure 1). Guilan and Mazandaran have a humid temperate climate with enough annual precipitation. 105 black tea samples were obtained randomly from 105 farms in the two different regions of Lahijan and Guilan provinces. The amount of each sample was about 100 g.

The glassware containers used for analysis were washed with detergent and rinsed several times with tap water to remove absorbance due to detergent; then they were soaked overnight in 6 N HNO_3_ (Merck) solutions and finally rinsed with deionized water. All aqueous solutions and dilutions were prepared with ultrapure water (c). 5 g of each tea sample was weighted by a digital analytical balance (Mettler Toledo, Switzerland) with ± 0.0001g precision and then was added to 500 mL of boiling tap water and allowed to infuse for 5, 15 and 60 min. Then samples were filtered under vacuum (using a Whatman No.42 filter paper) to eliminate any turbidity or suspended substance. Liquors from the first, second and third infusions were analyzed for lead, cadmium and arsenic contents by Inductively Coupled Plasma Optic Emission Spectrometry End of Plasma (ICP-OES EOP, Spectroacros, Germany). The blank solution was prepared in similar way without black tea.

The purity of argon as carrier gas was 99.999% (grade 5), with a flow rate of 0.7 L/min for supplementary and Modified Lichte nebulizer and 13 L/min for coolant flow. The speed of 4 channel peristaltic pump was 60 rpm for 45 S in pre-flush condition and 30 rpm for analysis. The power level was adjusted on 1400 KW. Before quantitative analysis of samples, calibration curves of the desired metals were prepared using a series of diluted standard solutions. The recovery percentage, detection limits, and % R.S.D for triplicate measurements of the measured elements were 90%–95%, 0.3 ppb, and less than 5%, respectively. Statistical analysis of the obtained results was preformed by SPSS 18 and One Way ANOVA test was used; results are expressed by mean ± SD.

## Results

The results of Pb, Cd and As obtained from tea infusion samples in 5, 15 and 60 min are presented in Table 1. The mean ± SD level of Pb in 5, 15 and 60 min in infusion tea samples were 0.802 ± 0.63, 0.993 ± 0.66 and 1.367 ± 1.06 mg/kg of tea dry weight, respectively. The mean level of Cd in 5, 15 and 60 min in infusion tea samples were 0.135 ± 0.27, 0.244 ± 0.46 and 0.343 ± 0.47 mg/kg of tea dry weight, respectively. The mean level of As in 5, 15 and 60 min in infusion tea samples were 0.277 ± 0.27, 0.426 ± 0.40 and 0.563 ± 0.45 mg/kg of tea dry weight, respectively. Also results showed infusion time influenced upon the amount of Pb, Cd and As in infusion tea (P < 0.001), (P = 0.001) and (P < 0.001), respectively.

**Table 1:** **The levels of lead, cadmium and arsenic in different infusions (*n =*** **945)**

**Heavy Metals (mg/kg)**	**Infusion times (min)**											
	**5**				**15**				**60**			
	**Min**	**Max**	**Mean**	**SD**	**Min**	**Max**	**Mean**	**SD**	**Min**	**Max**	**Mean**	**SD**
Pb	0.00	3.28	0.802	0.63	0.00	2.95	0.993	0.66	0.01	8.16	1.367	1.06
Cd	0.00	2.02	0.135	0.27	0.00	3.88	0.244	0.46	0.01	3.04	0.343	0.47
As	0.00	1.15	0.277	0.27	0.00	3.09	0.426	0.40	0.01	3.72	0.563	0.45

Table 2 presents the amount of Pb content according to locations and infusion time. The maximum level of Pb was determined in Soumehe-sara, which the amount of this metal in infusion time 5, 15 and 60 min were 1.24 ± 0.37, 1.94 ± 0.52 and 2.27 ± 0.63 mg/kg, respectively. The minimum level of Pb was determined in Fuman, and the amount in infusion times of 5, 15 and 60 min were 0.424 ± 0.29, 0.534 ± 0.34 and 0.725 ± 0.45 mg/kg, respectively. Also results showed location influenced upon the amount of Pb in infusion tea samples (P < 0.001).

**Table 2:** **The levels of Pb (mg/kg) in different infusions of tea samples (*n *315)**

**Region**	**Sample size**	**Infusion time (min)**											
		**5**				**15**				**60**			
		**Min**	**Max**	**Mean**	**SD**	**Min**	**Max**	**Mean**	**SD**	**Min**	**Max**	**Mean**	**SD**
Amlash	30	0.32	2.03	1.08	0.62	0.41	1.57	1.10	0.38	0.48	8.16	2.192	2.17
Bazkiagorab	3	0.51	0.51	0.508	0.00	0.6	0.6	0.597	0.00	0.62	0.62	0.624	0.00
Fuman	24	0.00	0.76	0.424	0.29	0.01	0.85	0.534	0.34	0.05	1.25	0.725	0.45
Ketalom	60	0.00	2.38	0.535	0.63	0.00	2.91	0.732	0.76	0.01	3.73	0.999	0.90
Kelar Abad	18	0.15	1.52	0.917	0.51	0.26	1.71	1.166	0.49	0.87	2.35	1.457	0.48
Lahijan	45	0.06	3.28	0.937	0.79	0.34	2.92	1.102	0.66	0.44	2.85	1.317	0.64
Ramsar	3	0.41	0.41	0.415	0.00	0.59	0.59	0.594	0.00	0.63	0.63	0.632	0.00
Rasht	21	0.39	0.86	0.627	0.17	0.59	1.67	0.949	0.36	0.6	2.35	1.270	0.64
Shaft	12	0.36	0.62	0.496	0.13	0.58	0.81	0.688	0.10	0.6	1.31	0.919	0.30
Siahkal	48	0.23	2.82	1.211	0.76	0.25	2.95	1.455	0.91	0.44	4.32	1.938	1.15
Soumahesara	6	0.98	1.51	1.246	0.37	1.58	2.32	1.948	0.52	1.82	2.72	2.271	0.63
Tonekabon	45	0.06	1.33	0.708	0.44	0.22	1.43	0.865	0.43	0.35	2.35	1.208	0.60

Table 3 showed the amount of Cd content according to locations and infusion time. The maximum level of Cd was determined in Amlash, for which the amount of this metal in infusion times of 5, 15 and 60 min were 0.387 ± 0.41, 0.545 ± 0.41 and 0.597 ± 0.43 mg/kg, respectively. The minimum level of Cd was determined in Soumehe-sara, for which the amount of this metal in infusion times of 5, 15 and 60 min were 0.002 ± 0.00, 0.012 ± 0.001 and 0.591 ± 0.81 mg/kg, respectively. Also results showed location influenced upon the amount of Cd in infusion tea samples (P < 0.001).

**Table 3:** **The levels of Cd (mg/kg) in different infusions of tea samples (*n *315)**

**Region**	**Sample size**	**Infusion time (min)**											
		**5**				**15**				**60**			
		**Min**	**Max**	**Mean**	**SD**	**Min**	**Max**	**Mean**	**SD**	**Min**	**Max**	**Mean**	**SD**
Amlash	30	0.03	1.06	0.387	0.41	0.06	1.15	0.545	0.42	0.07	1.25	0.597	0.43
Bazkiagorab	3	0.02	0.02	0.016	0.00	0.02	0.02	0.019	0.00	0.03	0.03	0.028	0.00
Fuman	24	0.02	0.26	0.077	0.08	0.03	0.4	0.121	0.12	0.05	0.78	0.225	0.24
Ketalom	60	0.01	0.82	0.188	0.22	0.01	0.99	0.283	0.30	0.02	0.99	0.315	0.32
Kelar Abad	18	0.00	2.02	0.351	0.82	0.02	3.88	0.889	1.51	0.02	3.04	1.072	1.10
Lahijan	45	0.00	0.26	0.052	0.06	0.01	0.31	0.068	0.07	0.01	0.43	0.091	0.102
Ramsar	3	0.04	0.04	0.039	0.00	0.06	0.06	0.055	0.00	0.07	0.07	0.068	0.00
Rasht	21	0.00	0.57	0.203	0.21	0.01	0.69	0.288	0.27	0.01	0.8	0.383	0.34
Shaft	12	0.00	0.22	0.106	0.11	0.02	0.36	0.157	0.16	0.03	0.63	0.258	0.26
Siahkal	48	0.00	0.1	0.028	0.03	0.00	0.22	0.064	0.05	0.01	1.17	0.165	0.26
Soumahesara	6	0.00	0.00	0.002	0.00	0.01	0.01	0.012	0.001	0.02	1.17	0.591	0.81
Tonekabon	45	0.00	0.23	0.045	0.06	0.01	1.06	0.226	0.35	0.06	1.76	0.511	0.56

Table 4 shows the amount of As content according to locations and infusion time. The maximum level of As was determined in Amlash, which the amount of this metal in infusion times of 5, 15 and 60 min were 0.552 ± 0.3, 0.709 ± 0.35 and 0.771 ± 0.21 mg/kg respectively. The minimum level of As was determined in Bazkiagorab, which the amount of this metal in infusion times of 5, 15 and 60 min were 0.011 ± 0.00, 0.014 ± 0.00 and 0.022 ± 0.00 mg/kg, respectively. Also results showed location influenced upon the amount of As in infusion tea samples (P < 0.001).

**Table 4:** **The levels of As (mg/kg) in different infusions of tea samples (*n *315)**

**Region**	**Sample size**	**Infusion time (min)**											
		**5**				**15**				**60**			
		**Min**	**Max**	**Mean**	**SD**	**Min**	**Max**	**Mean**	**SD**	**Min**	**Max**	**Mean**	**SD**
Amlash	30	0.22	1.10	0.552	0.30	0.28	1.3	0.709	0.35	0.57	1.16	0.771	0.21
Bazkiagorab	3	0.01	0.01	0.011	0.00	0.01	0.01	0.014	0.00	0.02	0.02	0.022	0.00
Fuman	24	0.01	0.39	0.159	0.18	0.02	0.59	0.243	0.21	0.02	0.79	0.343	0.28
Ketalom	60	0.00	0.62	0.198	0.21	0.01	0.78	0.299	0.23	0.01	0.86	0.389	0.26
Kelar Abad	18	0.00	1.15	0.410	0.43	0.04	3.09	0.974	1.09	0.56	3.72	1.411	1.19
Lahijan	45	0.00	0.59	0.213	0.15	0.02	0.7	0.353	0.18	0.04	0.79	0.459	0.20
Ramsar	3	0.22	0.22	0.224	0.00	0.29	0.29	0.292	0.00	0.43	0.43	0.43	0.00
Rasht	21	0.00	0.57	0.155	0.20	0.00	0.59	0.200	0.22	0.05	0.6	0.247	0.19
Shaft	12	0.00	0.42	0.207	0.17	0.00	0.57	0.321	0.24	0.01	0.6	0.377	0.27
Siahkal	48	0.02	0.65	0.218	0.19	0.08	0.77	0.396	0.23	0.24	1.04	0.644	0.24
Soumahesara	6	0.36	0.57	0.465	0.14	0.52	0.97	0.745	0.31	0.59	1.02	0.806	0.31
Tonekabon	45	0.02	0.97	0.404	0.37	0.02	1.18	0.513	0.41	0.1	1.36	0.661	0.44

## Discussion

The results showed that the maximum and minimum Pb of lead was determined in Soumahesara and Fuman, respectively. Also the maximum and minimum level for Cd was observed in Amlash and Soumahesara and the maximum and minimum level of As was determined in Amlash and Bazkiagorab. This study showed locations and infusion time influenced upon the amount of these metals (P < 0.05). The concentration of Pb in tea samples was lower than the permissible level (1 μg/kg) given by Iranian Ministry of Health. The amount of Pb level for Iranian’s tea samples were acceptable compared with limit prescribed by other countries such as China (5 μg/kg), India (10 μg/g) and Thailand (10 μg/g). The concentration of Cd in tea samples was lower than the standard limited value in Iran (10 mg/kg). The concentration of As in tea samples varied by locations and infusion time, that is below than that set as the standard maximum values by Iranian Ministry of Health (As 150 μg/g). All samples contained As at levels below by set the standard maximum values (4 mg/kg) [18].

The arsenic main sources of these heavy metals in tea samples are their growth media, such as soil. When fertilizers are applied to soils in tea cultivated area, heavy metals can be up taken by the plants. Soil contamination usually can be attributed to industrial, agricultural and urban activities. Table 5 shows the amount of Pb, Cd and As in tea samples from India, Turkey, Thailand, Taiwan, Pakistan, China, Ethiopia, Saudi Arabia, Iran and Nigeria [4,19-28].

**Table 5:** The amount of Pb, Cd and As (mg/kg) in different tea samples from other countries

**Ref.**	**Country**	**Year**	**Pb**	**Cd**	**As**
23	Iran	2005	0.531 – 5.3	0.038 – 0.272	---------------
5	Iran	2010	Non-detectable	Non-detectable	Non-detectable
4	Saudi Arabia	2008	0.3 – 2.2	0.3 – 2.2	--------------
10	China	2005	------------	0.056	0.28
6	China	2007	0.198 – 6.345	--------------	--------------
24	Pakistan	2006	0.25 – 4.75	Non-detectable	------------
3	Egypt	2008	0.03 – 0.76	------------	-------------
21	Turkey	2004	< 8 – 27.3	< 1 – 3	--------------
18	Thailand	2006	0.0237	0.0071	0.00084
26	India	2008	0.81 ± 0.32	0.14 ± 0.06	--------------

Tea samples from north of Iran do not seem to be heavily contaminated with Pb, Cd and As. Tea samples from north of Iran do not seem to be heavily contaminated with Pb, Cd and As. Since the tea cultivation lands in north of Iran provide much of black tea being consumed in Iran and the exported amount to other countries, it is recommended all elements and compounds in tea infusions to be analyzed for safe consumption of black tea. In order to be in safe side, it is recommended that tea to be prepared with heavy metal free and very low fluoride waters and preferably bottled water [29].

## Conclusion

After drinking water, tea is the most consumed beverage in Iran. In this regard health aspects related to tea is very important and therefore consumers should be very confident on the absence of any pollutants in black tea. The presence of any variations in metal contents in this study could be as a matter of the differences in methods of storage, tea leaf processing and the difference in soil metal concentrations. In addition, application of fertilizers causes increase of heavy metals in the cultivated soil.

In this study concentrations of lead, cadmium and arsenic were measured in black tea samples from different locations in different infusion times. It was deduced from the study that transfer of metals from black tea to brew depends on both locations and infusion times. The levels of lead, cadmium and arsenic were lower than the standards limits. Due to the lack of standards for all heavy metals in tea, it is recommended that the maximum allowable and safe concentrations to be established.

## Competing interests

The author(s) declare that they have no competing interests.

## Authors’ contributions

This study is a part of a research project. The study was directed by Dr. AHM who is the corresponding author and made the final preparation of article. Engineers SS, MG, and AM were engaged in sample preparations and laboratory work. Dr. MY helped on statistical data analysis and engineer SN as ICP expert performed the experiments by this device. The overall implementation of this study including the design, sample collection and preparations, laboratory experiments, data analysis, and manuscript preparation was performed by the corresponding author and the above team. All the authors have made extensive contribution into the review and finalization of this manuscript. All authors have read and approved the final manuscript.
